# Exploring Grassroots Indicators for Pandemic Prevention, Preparedness, and Response: A Systematic Narrative Review

**DOI:** 10.34172/ijhpm.8886

**Published:** 2025-12-27

**Authors:** Million Tesfaye Eshete, Pami Shrestha, Charmaine Ang, José María Valderas, David L. Heymann, Anders Nordström, Kelley Lee, Alex Cook, Clare Wenham, Pablo Perel, J. Jaime Miranda, Alberto L. Garcia-Basteiro, Helen Clark, Helena Legido-Quigley, Eivind Engebretsen

**Affiliations:** ^1^Centre for Sustainable Healthcare Education, Faculty of Medicine, University of Oslo, Oslo, Norway.; ^2^National University Health System, Singapore, Singapore.; ^3^Imperial College London, London, UK.; ^4^Saw Swee Hock School of Public Health, National University of Singapore, Singapore, Singapore.; ^5^London School of Hygiene and Tropical Medicine, London, UK.; ^6^Department for Global Public Health, Karolinska Institutet and Centre for Resilent Health, Stockholm School of Economics, Stockholm, Sweden.; ^7^Faculty of Health Sciences, Simon Fraser University, Vancouver, BC, Canada.; ^8^London School of Economics and Political Science, London, UK.; ^9^CRONICAS Center of Excellence in Chronic Diseases, Universidad Peruana Cayetano Heredia, Lima, Peru.; ^10^Sydney School of Public Health, Faculty of Medicine and Health, The University of Sydney, Sydney, NSW, Australia.; ^11^Centro de Investigação Em Saúde de Manhiça (CISM), Manhica, Mozambique.; ^12^Barcelona Institute for Global Health (ISGlobal), Barcelona, Spain.; ^13^The Helen Clark Foundation, Auckland, New Zealand.; ^14^George Institute for Global Health UK, London, UK.

**Keywords:** Pandemic Preparedness, Health Security, Grassroots Indicators, Community Engagement, Participatory Approaches, Bottom-up Approach

## Abstract

**Background:**

The COVID-19 pandemic has revealed how conventional top-down, expert-driven indicators often fail to align with local community realities, marginalising their perspectives, concerns, knowledge, and narratives. However, the limitations of pandemic-related and global health security indicators are not unique but reflect recurring patterns across major social metrics. In response, an alternative paradigm advocates for grassroots-inclusive approaches to developing indicators. Our objective is to assess how and why grassroots-inclusive approaches complement top-down approaches to developing indicators, and to synthesise their theoretical and practical contributions to public health.

**Methods:**

We conducted a scoping review in accordance with the Preferred Reporting Items for Systematic Reviews and Meta-Analyses extension for Scoping Reviews (PRISMA-ScR) guidelines. We systematically searched six databases (MEDLINE, Embase, CINAHL, Web of Science, Scopus, and PsycINFO), as well as Google Scholar, to identify relevant articles published from their inception to September 1, 2024. We included peer-reviewed articles, opinion pieces, and book chapters, narratively synthesising their findings.

**Results:**

This review included 43 studies from various disciplines. Across these studies, communities co-produced indicators through participatory workshops, interviews, and consensus exercises in areas such as environmental sustainability, disaster resilience, public health, well-being, and local development. The reported strengths included greater local relevance, community ownership, and accountability, alongside challenges in sustaining participation, integrating into top-down systems, and addressing data gaps. Notably, no study applied grassroots-inclusive indicators to health security or pandemic preparedness.

**Conclusion:**

Despite retrieving and analysing articles from various disciplines, no study has specifically applied grassroots-inclusive indicators to health security or pandemic preparedness. However, the evidence clearly shows that it is both feasible and practical to integrate expert and non-expert perspectives when developing indicators.

## Introduction

 Reflecting on the COVID-19 pandemic, a central paradox is the gap between how preparedness was measured before the crisis and how countries actually responded. While several global monitoring and evaluation frameworks were in place to assess pandemic preparedness and response, outcomes during the COVID-19 pandemic revealed patterns that were, at times, unexpected. In some cases, countries assessed as highly prepared experienced high mortality and significant challenges, while others assessed as less prepared demonstrated resilience.^1^ One critical omission in these indicators was the lack of attention to factors such as public trust in government, interpersonal trust, and corruption.^1^ In this paper, we argue that such omissions point to the need for indicators that are grounded in grassroots perspectives, which we later refer to as Grassroots Indicators (GRIs).

 We contend that this conflict between preparedness measures and actual health outcomes represents more than a simple omission of specific variables; it reflects a fundamental misalignment between the standardized metric and the complex reality it aims to represent. As Etzioni and Lehman articulated, this is a problem of “fractional measurement,”^2^ where operational indicators capture only a narrow slice of the broader social concept. This limitation is not unique to pandemic preparedness and response indicators. It applies to any class of *social indicators*.^3^ Often defined as statistics of direct normative interest that facilitate concise, comprehensive, and balanced judgments about the condition of major societal facets,^4^ social indicators share a defining trait: *They are predominantly crafted and implemented by experts on behalf of the populations they represent*.^5,6^ This expert-driven orientation often introduces blind spots. For instance, political leaders and economists have criticised gross domestic product for neglecting qualitative dimensions of growth, environmental degradation, and other dimensions that make life worthwhile.^7,8^ Similar critiques apply to other widely used, expert-designed indices such as the human development index,^9,10^ the *World Happiness Report*’s life-evaluation ladder,^11,12^ and the global health security index.^13^

 These critiques do not suggest that such indicators are without value.^6^ Instead, they highlight the difficulty of capturing complex, qualitative, and context-specific aspects of social life and social context through standardised metrics alone.^6^ While conventional, expert-driven indicators are useful for ensuring consistency and comparability across contexts, they may fall short in fully reflecting local realities, priorities, and knowledge systems.^5^ Much indicator work proceeds within what Kuhn terms normal science, which stabilises methods and success criteria within a professional scientific community.^14^ However, this can be limiting, as indicators may function as instruments of power that render some voices legible while marginalising others.^15^

 Given these contexts, this review aimed to synthesise evidence as a background paper for the National University of Singapore–Lancet Pandemic Readiness, Implementation, Monitoring, and Evaluation Commission.^16^ This commission was tasked with the reconceptualisation and monitoring of pandemic preparedness. Within the relevant literature, the development of grassroots inclusive indicators is variably framed as a participatory paradigm, community-based metrics, or neighbourhood-level metrics, among others. Here, we refer to these as GRIs. This review primarily aimed to examine the methodologies, conceptualisations, operationalisations, and practice of GRIs in the literature. We use GRIs to denote not only a local geographical scale, but also to describe the bottom-up power dynamic of their creation. To our knowledge, no prior study has undertaken such an analysis. The results are expected to provide new insights into the long-standing discourse on the evolution of social indicators over the past two centuries, informing future policy and practice.^8^

## Methods

 The research questions and objectives pursued in this review, as detailed below, lend themselves to a methodology that integrates both narrative and systematic reviews. As Collins and Fauser suggested,^17^ “An infusion of systematic review methods would strengthen narrative reviews, and in turn, systematic reviews could benefit from the strengths of narrative reviews.” While systematic reviews offer strength through rigorous, algorithmic methods, this rigidity can pose limitations when addressing questions requiring a broader, more interpretive approach. When tracing a concept’s historical evolution or crafting a nuanced narrative, forcing a systematic review risks losing the narrative thread.^17^ Nevertheless, whenever possible, this study adhered to the Preferred Reporting Items for Systematic Reviews and Meta-Analyses extension for Scoping Reviews (PRISMA-ScR) checklist.^18^

 However, the commonly employed structure for systematic reviews – the PICO (patient or population [P], intervention [I], control [C], and outcome [O]) framework and related variants^19^ – cannot be applied in this study for several reasons. Firstly, our population is highly diverse, spanning various nations, cultures, and settings. Secondly, while our interventions and controls are clearly defined (GRIs and conventional indicators, respectively), the outcomes vary across populations (eg, indicator development, identification, evaluation, and implementation). Limiting the review to a specific discipline, population group, or outcome to reduce heterogeneity would unfairly exclude important interdisciplinary and transdisciplinary knowledge relevant to this study. The approach adopted to address the challenges of abstracting and translating knowledge across disciplines is detailed in the following sections.

 We systematically searched six literature databases (MEDLINE, Embase, CINAHL, Web of Science, Scopus, and PsycINFO), as well as Google Scholar, employing forward and backward citation chasing to identify seminal papers. Seminal was defined as works that (*i*) first articulated or formalized a relevant concept/measure and (*ii*) were highly influential (top decile of forward citations within their publication decade in our corpus or repeatedly recovered across ≥2 citation chains). The searches covered the period from database inception to September 6, 2023 (the date of the last primary search). The search terms were customised to each database, as detailed in Supplementary file 1. We included peer-reviewed articles, opinion pieces, and relevant book chapters, and excluded conference abstracts and non-English papers.

 To capture the latest studies, we repeated our search of the six databases, covering the period from September 7, 2023 to September 1, 2024, using the original search terms and filters without modification. We maintained the same inclusion and exclusion criteria as those used in the original search to ensure methodological consistency. The updated search identified an additional 130 records distributed as follows: MEDLINE (*n* = 20), Embase (*n* = 24), CINAHL (*n* = 6), Web of Science (*n* = 15), and Scopus (*n* = 65). However, none of the articles met the inclusion criteria during the screening process. The final search was conducted on September 1, 2024.

 We used Covidence, a web-based review management software, to manage references, screen articles, and extract important information from each study. During the initial screening, the primary focus was on identifying articles that included the following terms: grassroots, community-level, participatory, local-level, citizen-generated, and bottom-up indicators. These terms were selected through an iterative process of testing various search strategies. Reviewers (MTE, HLQ, and EE), who were experts in public health and interdisciplinary health modified the search terms using their expert judgment where required. Texts with uncertain relevance were advanced to full-text review.

###  Search Strategy

 Two reviewers (MTE and PS) independently screened the full texts, and any disagreements were resolved by consulting a third reviewer. The primary exclusion criterion was a lack of relevant information to address the research objectives outlined above. Eight articles were added through forward and backward citation tracking. No geographic restrictions were applied. After study selection, we mapped each included study’s locale to United Nations geoscheme regions for descriptive reporting of coverage.

###  Screening and Selection Criteria

 We examined articles published in peer-reviewed international journals, book chapters, and editorials. For inclusion, each text had to meet the following conditions: (*a*) clearly incorporate at least one of the following terms or their variations – “grassroots indicator,” “participatory indicator,” “local indicator,” “community level,” or “neighborhood”; (*b*) describe or define indicators that align conceptually with these terms; and (*c*) reference an established definition of GRIs or related work. Texts that used keywords such as “grassroots,” “participatory,” or “local” without linking them to pertinent indicator concepts were considered out of scope and excluded. Texts found to lack relevant ideas or methods for developing indicators during full-text review were also excluded. We tracked the count of texts featuring the specified keywords and uploaded all identified titles into Covidence. After eliminating duplicates, we evaluated titles and abstracts against our inclusion criteria (Figure).

**Figure:**
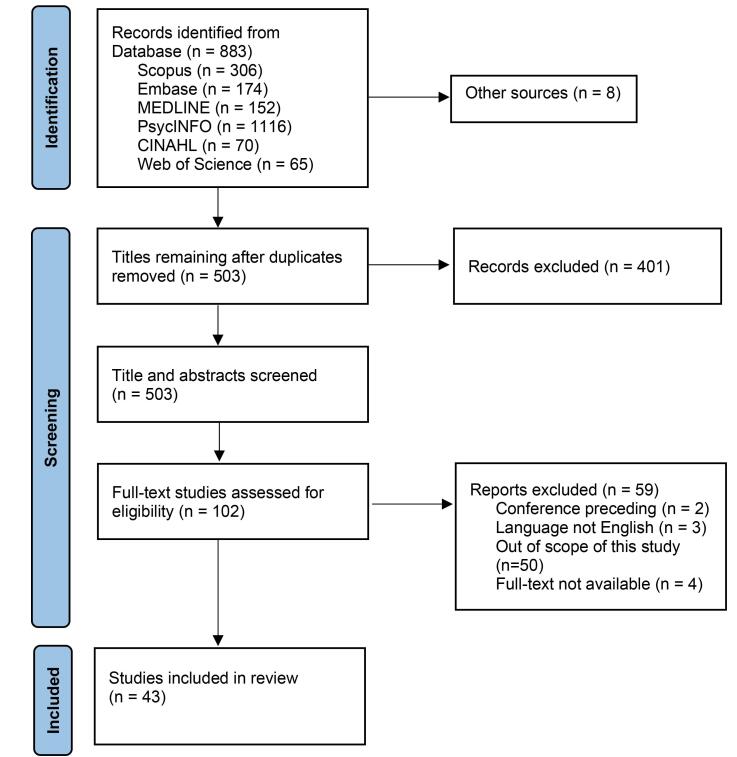
PRISMA 2020 flow diagram for the selection of studies. Adapted from: Page MJ, McKenzie JE, Bossuyt PM, Boutron I, Hoffmann TC, Mulrow CD, et al. The PRISMA 2020 statement: an updated guideline for reporting systematic reviews. *BMJ*. 2021;372:n71.

###  Data Extraction

 We developed and trialled a template for data extraction. Specifically, we extracted data related to the definitions of GRIs (if provided), methodologies used for their development, and the contexts and settings in which they were implemented. We extracted data on each study’s scope, main results, and evidence of the success or limitations of GRIs. The data extraction form was created using the Covidence software and was initially piloted by one reviewer (MTE). Then, two reviewers (PS and JT) performed the data extraction, which was validated by a third reviewer (MTE). The extracted information included publication year, study methods, objectives, conclusions, and key findings.

###  Data Analysis

 Analysing data across disciplines, especially from systematic reviews, is challenging, which is partly due to Kuhn’s notion of incommensurability. For Kuhn, incommensurability means that “Proponents of different theories (or paradigms, in the broader sense of the term) speak different languages – languages expressing different cognitive commitments that are suitable for different worlds. Their ability to grasp each other’s viewpoints is inevitably limited by the imperfections of the processes of translation and reference determination.”^20^ However, Kuhn’s notion of incommensurability is often misconstrued in several ways. One such mistake equates his notion of incommensurability with that of incomparability. We follow Kuhn in treating incommensurability not as incomparability but as a set of limits on translation across frameworks.^21^ To navigate heterogeneous accounts without forcing false equivalence, we conducted reflexive thematic analysis (RTA).^22^ RTA’s flexibility allowed us to address emergent patterns while remaining anchored to our analytical aims and stated theoretical commitments.^23^ We adopt a reflexive, interpretivist stance consistent with RTA. By theme, we mean a pattern of shared meaning underpinned by a central organising concept.^24,25^

 Our analysis was primarily inductive, involving open coding to let themes emerge from the data. However, we also applied a deductive approach to ensure the themes we identified aligned with our research questions.^23^ The following Results section is structured in two parts. First, it examines the various definitions of GRIs and the fields in which they are applied. Then, it examines the populations these indicators target and the methodologies used to develop them.

## Results

###  Definitions and Disciplinary Contexts

 Grassroots, participatory, citizen, and neighbourhood indicators can be considered a “family resemblance” (in Wittgenstein’s sense), united by their shared emphasis on integrating knowledge from local or indigenous communities or non-experts into their development.^26^ However, each places slightly different emphasis on specific aspects. For instance, Hambly defined GRIs as measures “formulated by individuals, households, and communities, and derived from their local systems of observation, practice, and indigenous knowledge.”^27^ In contrast, Sandoval and Rongerude used the term “participatory indicators” and emphasised their ability to validate community narratives and drive problem-solving.^28^ Fundamentally, these indicators challenge conventional top-down methods by prioritising community input, empowering local actors, and valuing non-traditional knowledge sources.

 Indicators possess both ontological and epistemological dimensions,^29^ which are weighed differently in different definitions. Ontologically, GRIs aim to reflect local *realities.*^30^ Using the COVID-19 pandemic as a case example, this could mean identifying and measuring factors that contributed to successful responses at the local level, such as the establishment of neighbourhood-level mutual aid networks, assisting vulnerable community members with essential tasks, such as grocery deliveries, prescription pickups, and other necessities, during quarantine or isolation periods.^31,32^

 Epistemologically, GRIs are fundamentally defined through processes of co-creation, implying that they are developed collaboratively with direct involvement from the communities they intend to represent. This collaborative approach ensures that indicators align closely with the lived experiences of community members and reflect their values.^28^ Table 1 presents the definitions of each term provided by the authors.

**Table 1:** Examples of Definitions and Contexts Provided by the Authors

**Reference**	**Author (Year)**	**Author Definitions**	**Context**	**Terms Used**	**Implication**
^3^	Bauer (1966)	“… statistics, statistical series, and all other forms of evidence … that enable us to assess where we stand and are going with respect to our values and goals, and to evaluate specific programs and determine their impact.”	How space exploration has sparked considerable public discussion and apprehension regarding its potential extensive impact on society.	Social indicator	Indicators as statistics for learning changes in the real world.
^33^	Henderson (1989)	“The now widely shared insight that sustainable development must begin local/y from the grassroots and basic agriculture, rather than the failed trickle-down model of industrialism.”	Grassroots inclusive development and indicators based on these grassroots realities.	GRI	The problem lies in over-aggregating statistical data, which ignores social costs and non-monetary sectors.
^27^	Hambly (1996)	“Measures or signals of environmental quality or change formulated by individuals, households and communities, and derived from their local systems of observation, practice and indigenous knowledge.”	The term “environment” is defined comprehensively, encompassing social, economic, ecological and cultural dimensions. This broad perspective aims to move beyond the narrow, sector-specific focus typically found in traditional environment and development indicators.	GRI	GRI valuing indigenous knowledge.
^34^	Sawicki and Flynn (1996)	“Neighbourhood generally implies an area smaller than a municipality but more than a few city blocks.”	“First, the indicators must be formulated in a participatory process that includes residents and experts.” “Second, the indicators must be capable of affecting citizen action and public policy-making.”	Neighbourhood indicator	Fluidity of neighbourhood indicators as non-human entities and neighbourhood indicators as human entities.
^35^	Nazarea (1998)	“Grassroots indicators attempt to understand local populations through their own internally defined standards, many of which are qualitative, non-monetary, non-material, and long-term and which often define what makes life, society, and the environment worthwhile.”	Cultural values shape a range of considerations, such as emotions, aesthetics, spirituality, ethics, beliefs about the universe, and the desire to leave a legacy for future generations, all of which directly influence resource management and the practice of sustainable agriculture.	GRI	GRI valuing indigenous knowledge.
^36^	Eckerberg and Mineur (2003)	“If the changes in the indicator value are directly linked to individual actions, it is classified as a citizen-oriented indicator.”	“Citizen-oriented indicators with a prime purpose of starting a dialogue with the citizens.”	Citizen-oriented indicator	Technocratic articulation of indicators.
^30^	Chambers (2010)	“…participatory methodologies (PMs) can be defined as combinations of approaches and methods through which people are facilitated to do things themselves.”	The paradigm of goals, designs, and indicators is negotiated, evolving, and emergent.	Participatory indicators	Indicators and the reality of the poor people, in the context of adaptive pluralism.
^28^	Sandoval and Rongerude (2015)	“Indicators that involve community participation create a space for sharing local stories, which in turn helps to pinpoint issues within the neighborhood.”	Towards indicators that acknowledge community narratives as credible information, support communities in tackling challenges, and convert local issues into steps for improvement.	Participatory indicator	Indicators valuing the issue of participation, empowerment and power.

Abbreviation: GRI, Grassroots Indicator.

 Most of the reviewed texts associated GRIs with the concept of scale. However, the term “scale” is often used interchangeably—and ambiguously—with “level,” despite warnings from some scholars about the importance of distinguishing between these terms.^37^ GRIs are often regarded as the lowest tier in the analytical hierarchy and are thus seen as more proximate to the lived realities of local populations.^33,38^ Hence, they have the potential to provide a detailed and authentic reflection of the phenomena they aim to measure.^38^ Unlike conventional indicators, which often involve experts imposing external concepts, frameworks, and vocabularies, GRIs prioritise the values and concerns of the communities they represent.^27,28^ Moreover, it has been argued that many conventional indicators remain fixed at the national and global level.^39,40^ This mismatch can undermine decision-making; national-level data may not accurately reflect grassroots realities, just as regional decisions falter when based solely on national indicators.^40^ Specifically, in public, global, and planetary health, lower-level indicators—those at the primary healthcare (PHC) level—have been identified as appropriate for interventions and measurement.^39^ However, they are often ignored.^41^

 GRIs have been applied to diverse disciplinary contexts. Sustainability stands out as the most common context,^42-47^ including closely related topics such as environmental health,^48^ biodiversity conservation,^49^ and disaster resilience.^50^ Health-related applications are also significant, spanning public health,^51,52^ community health,^53^ and specific issues, such as human immunodeficiency virus (HIV)^54^ and healthcare systems.^52,55^ Beyond these applications, GRIs have been applied to community-focused efforts, such as empowerment^28^ and well-being,^56^ as well as in economic domains,^57^ including desertification,^27^ eco-efficiency,^40^ and coastal management.^58^ Interestingly, despite this diversity, no studies have yet utilised GRIs in the context of health security or pandemic preparedness, a gap that suggests opportunities for future research.

###  Populations and Methods

Table 2 provides details on the studies, including their target population, methods, and key findings. Most studies were conducted in North America, particularly the USA and Canada,^28,33,42,44,53,59^ followed by Europe, with contributions from countries such as France,^56^ Germany,^51^ and Finland.^40,55^ Other prominent regions were Latin America (eg, Mexico^43^ and Brazil^58^), South Asia (eg, India^54^ and Bangladesh^60^), Southeast Asia (eg, the Philippines^50^), Oceania (eg, Australia^61^), and Africa (eg, Tanzania^57^ and Botswana^62^).

**Table 2:** Design, Objectives, Methods, and Main Results of the Included Studies

**Reference**	**Author (Year)**	**Record Type**	**Setting**	**Methodology**	**Scope/Objective**	**Main Finding/Comments**	**Indicators Developed**
^63^	Asare-Kyei et al (2015)	Peer reviewed	Ghana, Burkina Faso, and Benin.	A multi-stage, multi-methodology participatory process	To pinpoint specific sets of indicators, validated at local and national levels, that can be used to measure the risk posed by natural hazards.	Some indicators considered important by local experts were omitted at the national level.	Yes
^29^	Canagarajah and De Costa (2016)	Peer reviewed	NA	Review and debate.	To examine changing perspectives on scalar analysis in linguistics, propose future research directions, and address its epistemological and ontological challenges.	Explores changing perspectives on scalar analysis in linguistics and outlines potential areas for future research. Additionally, addresses epistemological and ontological challenges associated with scalar analysis.	No
^59^	Cheadle et al (2000)	Peer reviewed	USA	The modified Delphi technique.	To develop community-level indicators that evaluate the community-based programme and prevention of cardiovascular disease.	Identified how community-level indicators can be applied and are useful in preventing cardiovascular disease. These indicators are categorised into four key areas: behavioural outcomes, environmental modifications, information dissemination, and policy.	Yes
^64^	Chambers (1995)	Peer reviewed	NA	Review and overview.	To determine how experts’ generalised and simplified perspectives on poverty diverge from the lived experiences of impoverished individuals.	This work presents new perspectives on concepts such as poverty, wealth, well-being, employment, and livelihood. It also champions altruism and systemic changes to enable poor individuals to analyse and articulate their own needs.	Yes
^65^	Chambers (2007)	Grey literature	NA	Review and overview.	To examine the differences between participatory methods of monitoring and evaluation and classical methods.	The results challenge current frameworks, practices, and research on indicators, advocating instead for the use of participatory data and methods.	No
^6^	Cobb and Rixford (1998)	Grey literature	USA	Descriptive.	To articulate the lessons learnt from the social indicator movement.	Presents 12 crucial lessons, each illustrated with a real-world example, offering new insights into poverty, wealth, well-being, employment, and livelihood.	No
^52^	Daniel et al (2009)	Peer reviewed	Canada, Australia, and Aotearoa/New Zealand	A sequential multi-methodology: literature review, domain identification, format determination, pre-testing for validity, initial tool revision, pilot testing, and final revision.	To create criteria and an assessment instrument that enables healthcare providers, Indigenous community members, decision-makers, and academics to evaluate the relevance and appropriateness of current health and social metrics.	The indicator demonstrated strong internal reliability and high inter-rater agreement. It thoughtfully integrates both cultural and scientific considerations, enabling stakeholders (researchers, funding agencies, and community members) to identify relevant indicators for evaluating the effectiveness of interventions.	Yes
^47^	Dawodu et al (2021)	Peer reviewed	NA	Literature review and applied established participation-evaluation frameworks.	To identify the advantages and disadvantages of incorporating participatory indicators when evaluating neighbourhood sustainability assessment tools.	Many of the examined tools failed to truly boost participation. Participatory indicators are often downplayed compared to non-participatory ones, and simply consulting the public does not guarantee their feedback will be put into practice.	No
^36^	Eckerberg and Mineur (2003)	Peer reviewed	Sweden	Case studies.	To evaluate the design of five distinct local sustainability indicator frameworks in Stockholm and Sundsvall, Sweden, focusing on their inclination towards expert or citizen participation.	Authentic public involvement in sustainability indicator schemes often wanes after the indicators are established, revealing a persistent gap between the public and policy-makers. The findings highlight the difficulty of actively engaging citizens in implementing sustainability initiatives through these schemes.	No
^2^	Etzioni and Lehman (1967)	Peer reviewed	NA	Overview and debate.	To assess the shortcomings of social metrics in informing societal planning and the broader challenges inherent in quantifying social concepts.	When working with social indicators, it is crucial to consider several key concepts, including the multidimensional nature of measurements, the difference between means and goals, concept reduction, and the understanding that indirect proxies might not fully represent their intended targets.	No
^58^	Fontalvo-Herazo et al (2007)	Peer reviewed	Brazil	Three-step approach involving stakeholder engagement to prioritise issues, followed by designing a hierarchical indicator framework, and finally filtering and selecting indicators.	To address the core challenge of engaging stakeholders, particularly local users, in the creation of indicators. To propose a participatory method for developing coastal management indicators with direct input from coastal communities, and then demonstrate and evaluate this approach in Brazil’s Bragança coastal zone.	Stakeholders initially identified and then prioritised key environmental, social, governance, and economic sustainability factors in workshops, which led to a hierarchical indicator framework – with 4 principles, 11 criteria, and 35 indicators – that effectively tracks change and informs local integrated coastal management policies.	Yes
^46^	Fraser et al (2006)	Peer reviewed	Canada, Botswana, and Guernsey (UK).	Case studies and participatory methods.	To synthesise findings from three intentionally diverse case studies that moved from top-down approaches to deeper community engagement as a basis for improving environmental monitoring and management.	Empowering communities by involving them in the choice of indicators is often absent from standard development. For policy-makers to deem these indicators relevant, it is crucial to embed stakeholder-driven processes within formal decision-making. Adaptability in selecting monitoring and decision scales is also essential.	Yes
^27^	Hambly (1996)	Grey literature	Kenya, Tanzania, Zimbabwe, and Uganda	Book chapter.	To define, develop, and implement a GRI in the context of desertification.	Explores GRIs and scientific indicators. It also examines research methods for identifying and validating GRIs.	Yes
^33^	Henderson (1989)	Peer reviewed	NA	Descriptive.	To summarise why social indicators, measures of life quality, and innovative accounting techniques are essential and how they are utilised. To explain how these can be used to design and implement mutual development initiatives that prioritise grassroots participation, cultural diversity, and global sustainability.	To understand rapid, irreversible changes, we need more flexible and dynamic modelling, possibly drawing from chaos theory.	No
^66^	Hermans et al (2016)	Peer reviewed	The Netherlands	The approach integrates examining documents, observing participants, and conducting detailed interviews with important sources of information.	To examine the process by which community-driven sustainable farming innovations are expanded and how this expansion affects the nature of these innovations as they operate at various scales and levels.	This study employed a five-tier framework to analyse a dairy farming and landscape management innovation. It found that upscaling and outscaling fundamentally alter the structure and meaning of grassroots innovations.	No
^67^	Howes and Chambers (1979)	Peer reviewed	NA	Synthesis of the discussions and insights shared during the workshop focused on the traditional knowledge of Indigenous peoples.	To thoroughly examine ITK and compare it with institutional science.	This study details the utilisation of this underused knowledge for generating, assimilating, and transmitting knowledge, and introduces new ITK elicitation methods.	No
^68^	Innes (1988)	Peer reviewed	USA	Commentary.	To examine the links between data and decision-making.	Data utilisation is intrinsically linked to its production and interpretation. The findings emphasise that data’s core purpose goes beyond understanding, consistently linking to specific decisions.	No
^60^	Islam et al (2021)	Peer reviewed	Bangladesh	Literature review, extensive interviews, structured workshops, team-based discussions, and hands-on testing of the indicator framework.	To use a bottom-up, community-driven participatory model, involving local residents in defining sustainability objectives, choosing indicators, and fully owning the system’s ongoing operation.	Developed a comprehensive 16-step framework for indicator creation, ensuring indicators were formulated from the local community’s perspective.	Yes
^69^	Land (2002)	Peer reviewed	USA	Revision databases and population surveys.	To assess the accessibility of data at the state and local levels for monitoring the objectives and leading health indicators of Healthy People 2010.	This study quantified the inconsistency of data sources available for monitoring relevant health goals at the state and national levels.	No
^55^	Mäntyranta et al (2004)	Peer reviewed	Finland	The approach involved a comparative needs assessment, using existing healthcare needs assessments, healthcare provision databases, and relevant information systems.	To merge data on local healthcare demands, drawn from national records and other resources.	This study concludes that indicators should be trustworthy, dependable, and simple to understand. They must also: (*a*) capture a health factor influencing many people, and (*b*) act as a resource for evaluating needs and shaping community healthcare and public health initiatives.	Yes
^8^	Mayoux and Chambers (2005)	Peer reviewed	NA	Review and overview.	To present new findings and creative solutions to promote “reversing the paradigm,” asserting that community-involved techniques should take a primary role in most monitoring and evaluation.	Participatory methods are crucial for identifying relevant local indicators. They produce accurate quantitative data while capturing local priorities. These approaches also reveal diverse experiences and innovation potential, especially in terms of causality. They are also cost-effective for focused quantitative and qualitative studies, making it crucial to address related development challenges.	No
^45^	McAlpine and Birnie (2005)	Peer reviewed	Guernsey (UK)	Participatory approach.	To (1) establish shared strategic goals, (2) define common organisational policies to achieve these goals, and (3) facilitate effective resource allocation and management for policy implementation.	The project’s UN Agenda 21-driven stakeholder engagement faced initial disinterest due to past unfulfilled initiatives. It began with top-down preliminary objectives, which were then refined through stakeholder input. A key turning point was when a few stakeholders showed interest, enabling the refinement of indicators and broader community involvement.	Yes
^70^	Meadows (1998)	Grey literature	NA	Report from an interdisciplinary, diverse workshop.	To examine the concept of indicators and information systems in the context of sustainable development goals and practical implications.	A recommended model for sustainable development metrics, accompanied by illustrative examples.	Yes
^40^	Mickwitz et al (2006)	Peer reviewed	Finland	Participatory approach and life cycle impact assessment method based on decision analysis.	To monitor changes using economic-environmental ratio indicators and concurrently gain insights into the region’s social progress.	Ongoing communication is essential among the various stakeholders in the region to ensure indicators are utilised effectively in promoting sustainable development.	Yes
^35^	Nazarea (1998)	Peer reviewed	The Philippines	A case study employing a practical ethnoecological perspective and an adjusted form of the thematic apperception test.	To offer an approach to analyse culturally meaningful indicators and explore how people from diverse ethnicities, sex and age ranges differently interpret a given reality.	Context-sensitive indicators differ significantly from external ones, varying systematically in relation to socioeconomic and sociodemographic factors. Incorporating culturally relevant indicators can shape locally resonant development pathways, providing both short- and long-term benefits.	Yes
^71^	Newcomer (2000)	Peer reviewed	USA	A case study demonstrating the process of embedding survey results into an indicator system.	To illustrate how existing data can be arranged and used to benefit communities, health programmes, and distinct population segments.	When monitoring chronic conditions at the local level, indicators should be selected from databases that contain information on healthcare services. Local indicators should encompass multiple aspects related to healthcare provision.	No
^44^	Parkins et al (2001)	Peer reviewed	Canada	Community-engaged research conducted through workshops, a system for evaluating indicators, and a questionnaire.	To develop a regionally customised method for pinpointing social markers of community sustainability.	The findings emphasise the issue of “one-size-fits-all” approaches to community sustainability and that varying community perspectives on sustainability progress require tailored indicators for each.	Yes
^39^	Patrick et al (2019)	Peer reviewed	NA	Scoping literature review.	To determine current measures that establish a connection between human welfare and environmental conditions.	The Happy Planet Index, a blended metric devised by the New Economics Foundation, was viewed as an effective tool. It can be adjusted using data specific to a particular local or regional area.	No
^61^	Patrick et al (2022)	Peer reviewed	Australia	Community-driven research using a qualitative approach, featuring 17 interviews, 4 focus groups with 27 total participants, and a document review.	To develop a combined metric for local purposes, specifically an adapted version of the Happy Planet Index, computed as life expectancy times life satisfaction and an equity factor, divided by ecological footprint.	The One Planet approach can boost community engagement with planetary health. It is a valuable tool for advocating for integrated health/sustainability policies and justifying programmes based on shared benefits. It also allows local–national comparisons. Broadly, it helps break down disciplinary silos, enhancing health promotion and planetary health partnerships.	No
^62^	Reed et al (2008)	Peer reviewed	Botswana	Participatory and ecological methods.	To recognise and assess metrics connected to the sustainability of the environment.	Metrics created via combined community-involved and ecological studies are well-known to pastoral communities and can be applied by local residents without expert instruction. However, some indicators employed by pastoralists do not accurately measure land degradation. Nonetheless, local wisdom offers a broader perspective compared to many indicator lists available in academic or official sources.	Yes
^51^	Rohrbein et al (2023)	Peer reviewed	Germany	Participatory approach.	To develop health indicators at the neighbourhood level for tailored decision-making.	Successfully developed and implemented the indicators. The unavailability of data for some relevant indicators was acknowledged as a limitation.	Yes
^56^	Roy et al (2015)	Peer reviewed	France	Quantitative questionnaire, qualitative interviews, and triangulation.	To formulate a conceptual model along with social indicators to evaluate the quality of life in sustainable territories.	This study established a methodological and theoretical framework for indicator development. Constructing policy-relevant indicators requires a collective and participatory approach, which is essential for a pertinent public policy theory.	No
^48^	Salgado et al (2020)	Peer reviewed	Portugal	A participatory strategy, carrying out semi-structured interviews, and systematically reviewing relevant literature and databases.	To choose indicators to guide the monitoring and evaluation of environmental health.	A high level of participation demonstrates the usefulness of the participatory approach in validation. The process uncovered missing information in indicators related to noise and movement, as well as new housing concerns that require further investigation.	Yes
^28^	Sandoval and Rongerude (2015)	Peer reviewed	USA	Participatory approach.	To develop formative indicators.	Community-driven indicators strengthen communities and aid in problem identification. In a local context, it is possible to establish a process shaped by residents themselves by employing indicators that are easy to comprehend and relevant to the situation.	No
^43^	Santana-Medina et al (2013)	Peer reviewed	Mexico	Participatory approach.	To investigate a sustainability assessment conducted with community participation in a locality situated within a natural reserve. To deliver theoretical insights and practical tools to enhance the assessment of sustainability from a societal viewpoint.	A four-step process was utilised to identify and select sustainability metrics: describing the system, setting objectives, choosing indicators, and evaluating progress. Then, the selected indicators were organised according to the three main aspects of sustainability and how they interrelate.	Yes
^34^	Sawicki and Flynn (1996)	Peer reviewed	NA	Literature review.	To assess conceptual and methodological issues.	Quantitative indicators need a clear policy purpose. Geographic indicators are crucial, more so than subject-area ones, as policy is inherently geographic and neighbourhoods influence lives. Neighbourhood and resident well-being indicators should be distinguished. Finally, indicators are most useful when they are not bundled in a single overall index.	No
^72^	Sherry et al (2005)	Peer reviewed	Canada	Based on archival records from government sources and pertaining to local-level criteria and indicator efforts, used alongside three widely recognised frameworks for sustainable forest management.	To understand the differences between local interpretations of sustainability and those imposed from a global, centralised perspective. The study juxtaposed the standards and metrics used by Indigenous communities at the local level with three well-known models.	There are numerous frameworks for criteria and indicators in sustainable forest management, which differ in their intricacy and the extent to which they incorporate local contexts. According to the findings, community-derived definitions yield more granular criteria and indicators than those from national or global frameworks. Considering both community-specific and wider viewpoints is essential to ensure that sustainable management aligns with actual needs.	No
^54^	Shukla et al (2016)	Peer reviewed	India	Qualitative research (interviews and focus groups).	To explore how both personnel and donor representatives of non-government organisations interact with and perceive the indicators’ monitoring and evaluation frameworks.	The central issue is a disconnect between “real work” and indicators, with targets often met through unethical means. Indicators are deemed misleading, inflexible, and suffer from data manipulation and underuse, making their application an “empty ritual” without local resonance.	No
^42^	Sonntag (2010)	Peer reviewed	USA	Participatory approach and case study.	To develop an advanced sustainability indicator platform dubbed the B-sustainable Information Commons.	Indicator frameworks make a significant contribution to social learning. Testing the underlying assumptions of their traditional models is a necessary step in their evolution.	No
^49^	Soykan and Lewison (2015)	Peer reviewed	NA	Meta-analysis (synthetic approach).	To assess how reliable community-level metrics are for management purposes.	Metrics from biomass datasets showed greater consistency than those from abundance data. Community-level biomass also demonstrated more predictable differences between marine protected areas and control sites than abundance.	No
^53^	Stone et al (2010)	Peer reviewed	USA	Community-based participatory research.	To develop meaningful indicators.	Community behavioural health measures have continued to evolve, even in the face of deployment hurdles, overlapping initiatives, and unclear guidance on application.	Yes
^50^	Talubo et al (2022)	Peer reviewed	The Philippines	Combination of participatory Methods: semi-structured and expert interviews, and a web-based Delphi.	To create metrics that merge hands-on, practitioner-driven methods with rigorous statistical techniques.	Many indicators were identified for evaluating an island community’s disaster resilience. Creating novel approaches to assess island community disaster resilience is seen as a beneficial advancement, improving comprehension and putting the concept of resilience into practice.	Yes
^57^	Van Campenhout (2007)	Peer reviewed	Tanzania	Participatory wealth rankings through a large, multi-household representative sample survey.	To investigate the use of subjective wealth assessments in place of conventional financial metrics, such as income or spending, to evaluate micro-level poverty in remote, traditional rural communities.	Local residents’ insights into their neighbours’ circumstances provide a trustworthy ranking of household welfare, offering a substitute for problematic measures such as income or spending levels.	Yes

Abbreviations: ITK, Indigenous traditional knowledge; GRI: Grassroots Indicator; NA, not applicable; UN, United Nations.

 As highlighted in several key studies,^45,46,59,60^ the process of developing GRIs varied, including in identifying potential indicators through participatory methods, supplementing insights from existing literature, and establishing benchmarks and data sources in collaboration with community members. Community engagement and stakeholder involvement also varied. For example, Fraser et al^46^ described community empowerment in a project in Coastal British Columbia, Canada, by actively engaging a wide range of stakeholders, including local communities, industry representatives, government agencies, and environmental groups.

 Similarly, when creating indicators for heart health initiatives, Cheadle et al^59^ reported how experts from various sectors, including state health departments, universities, and government institutions, employed a consensus-building technique to incorporate different viewpoints. In efforts to make tourism in Boga Lake, Bangladesh, more sustainable, indicators were collaboratively developed with the community through methods such as group discussions, one-on-one talks, and interactive sessions, ensuring they were relevant, significant, and aligned with the community’s priorities.^60^ In contrast, on Guernsey, selection combined expert analysis and government priorities with community feedback.^45,46^

 Across the corpus, some studies directly involved local residents in indicator development,^28,43,44,46,51,52,56-58,60,62^ whereas others limited involvement to selected experts and institutional stakeholders, despite being labelled participatory.^40,48,61^ The selection of indicators also varied. Some case studies selected the final indicators based on consensus, while others prioritised feasibility and availability to facilitate selection and validation.^46,59^ Nonetheless, most case studies adopted established criteria or attributes of good indicators that aided stakeholders in shortlisting the indicators to include.

 Implementation strategies for GRIs differed across studies. In some cases, such as coastal management in Brazil, GRIs were disseminated through implementation strategies that differed based on the project’s objectives and the community’s capacity.^58^ In others, such as tourism sustainability in Boga Lake, Bangladesh, the final set of indicators was implemented collaboratively with a community research team, empowering community members to take charge of their own development and advocate for their interests effectively in decision-making processes.^60^ Overall, what is common is community empowerment through the development of GRIs across various domains. Table 3 provides examples of steps involved in indicator development, and Box 1 provides an example of the application of GRIs in public health.

**Table 3:** Examples of Steps in Grassroots Indicator Development

**Reference**	**Author (Year)**	**Steps Involved in Indicator Development**	**Methodological Approach**	**Steps in Overall Indicator Development**
^60^	Islam et al (2023)	The community was actively involved in the indicator development process through participatory methods. Stakeholders, including local residents, tourism operators, and government officials, were engaged in discussions, focus groups, and consultations to ensure their perspectives were incorporated into the indicator.	The methodological approach prioritised community engagement from the outset. Participatory techniques, such as workshops, focus groups and interviews, were utilised to gather input from stakeholders. This approach ensured that the indicators developed were relevant, meaningful, and reflective of the community’s values and priorities.	The indicators were developed iteratively, with stakeholder involvement at each stage. The process included identifying potential indicators, supplementing them from the literature, prioritising areas, evaluating each indicator based on criteria, and establishing benchmarks and data sources. The final indicator is implemented collaboratively with a community research team.
^45^	McAlpine and Birnie (2005)	Community engagement and consultation are prioritised to ensure diverse perspectives are considered in indicator selection. Thorough public and private consultations gather insights from various stakeholders across social, economic, and environmental sectors.	Applied both bottom-up and top-down approaches for indicator selection. Initial indicators may be identified based on expert analysis, while others emerge from community feedback and consultation processes. The process emphasises flexibility and adaptation to changing community needs and data availability.	Indicators and priority areas were initially chosen based on expert analysis and government priorities. The community then engaged to refine them. Flexibility and adaptation are emphasised throughout the process to ensure ongoing relevance. The final indicators are disseminated annually for community engagement.
^59^	Cheadle et al (2000)	Various stakeholders, including health experts, were engaged in the Delphi process to ensure diverse perspectives and expertise.	A structured Delphi process allowed for expert consensus-building on potential indicators. Through multiple rounds of surveys and feedback, the indicators were evaluated based on their quality and feasibility, ensuring the selection of accurate and practical community-level indicators.	Using a structured Delphi method with programme evaluation and cardiovascular prevention specialists, the study identified behavioural risk indicators – diet, sedentary lifestyle, and smoking – and chose them according to stringent quality criteria (accuracy, relevance, consistency, and validity), practical feasibility (data access and affordability), and their comprehensive suitability for assessing community health initiatives.
^46^	Fraser et al (2006)	In Coastal British Columbia, Canada, the authors emphasised community empowerment throughout the indicator identification process. Stakeholder engagement involved various parties, including local communities, industry representatives, government agencies, and environmental groups.	The authors employed a combination of participatory processes and scientific analysis. Consultative processes involving technical committees and stakeholders were utilised to identify indicators. Scientific methods, such as data collection and analysis, also supported the evaluation and refinement of indicators.	Indicators were developed through collaborative efforts between researchers and stakeholders. Relevant indicators for measuring human and environmental well-being were identified through consultative processes and technical assessments. These indicators were selected based on their ability to address environmental concerns and support sustainable resource management practices.
^46^	Fraser et al (2006)	In the Kalahari Rangelands of Botswana, the authors emphasised community empowerment. Collaborative efforts between researchers and local communities ensured an inclusive and participatory indicator development process.	The authors used a sustainable livelihoods analysis framework to identify indicators. They gathered community input through interviews and focus group discussions.	Indicators were developed collaboratively through iterative discussions and scientific appraisals. The identified indicators were then evaluated based on accuracy, feasibility, and relevance to the local context.

**Box 1. Collaborative Approach to Building Local Health and Well-Being Indicator Systems**

*Study objective:* This study^51^ aimed todevelop neighbourhood-level urban health indicators for localised health promotion priorities, planning, and evaluation.

*Setting and indicator development process:* Conducted in Mannheim, Germany, this study’s indicator development began with extensive discussions with the city’s public health department. They chose a pilot neighbourhood without prior health promotion projects, where the local manager was highly interested in integrating health, and where a neighbourhood assessment tool was desired. The researchers used an iterative plan–do–check–act process, with active citizen stakeholder participation in selecting community-valued indicators, initially drawn from the literature.

*Validation of indicators:* After gathering feedback from the community and stakeholders, the researchers contacted data owners to confirm the availability of the desired data, both aggregated and non-aggregated. Any elements that did not meet the criteria or were unavailable but still deemed relevant were noted for potential future inclusion in the neighbourhood indicator. To ensure ease of use and updates, the researchers developed a tool with a visual interface, which then underwent beta testing and feedback sessions with various stakeholders.

*Implementation, outcome, and impact:* The process of enacting the plan began with conversations with the city’s health officials to determine who among the interested parties would use the tools, and their involvement was tracked. Working together to create and apply similar tools in various areas enables local participants to take an active role in identifying and reducing health differences within regions. Overall, the step-by-step, community-involved method of building the indicator system proved effective, despite obstacles such as the outbreak of the COVID-19 pandemic and a lack of specific data.

Several authors recommend systematically combining top-down and bottom-up approaches to ensure the accuracy of indicators.^62^ Some have developed guidelines for this process,^61^ while others have outlined detailed steps for integrating expert and grassroots perspectives.^70^ Methods for achieving this integration range from design frameworks to procedures for identifying locally defined indicators with community engagement,^44^ and criteria have been proposed to balance scientific and indigenous knowledge in community-level indicators.^52^

## Discussion

 This review identified 43 studies that illustrate how communities have co-produced indicators across various domains, including environmental sustainability, disaster resilience, public health, well-being, and local development. These studies employed participatory methods, including workshops, interviews and consensus exercises, to generate indicators reflecting local priorities.^44,48,50,58,60,70^

 Our findings indicate that co-produced indicators can result in improved local relevance, accountability, and ownership, while simultaneously challenging conventional assumptions about scale, power relationships ^28,40,45,68,70^ and the boundaries between expert and non-expert knowledge.^27,45,67^ However, despite the breadth of disciplines represented, no study applied these approaches to pandemic preparedness or health security, highlighting a major gap that must be addressed in future research.

 This review also contributes new knowledge to the long-standing debate on social indicators. This debate centres around four central themes regarding social indicators: (1) their purpose (prescriptive vs descriptive), (2) their development (deductive vs inductive), (3) the tension between bias and objectivity, and (4) their utility (academic tools vs governance frameworks).^6^ This review advances the second theme by adding a new dimension to the deductive-inductive axis: the participatory nature of indicators and contrasting bottom-up and top-down approaches. This contrast positions indicator development between normal and post-normal science.^14,73,74^

 In Kuhn’s definition of normal science, participation is limited to a well-defined community of professional peers rather than the public.^14^ In contrast, post-normal science advocates for the inclusion of extended communities and their knowledge when uncertainty is high and decisions are urgent.^72^ Pandemics are one of those domains where decisions are urgent and uncertainty is higher, making bottom-up approaches in indicator development a necessity. Consequently, the four lessons gained from this review are as follows.

 Firstly, determining the appropriate level for indicator development is a complex process. However, in practice, levels are sometimes chosen for convenience, with fitness for purpose secondary.^34^ Consequently, PHC levels are often overlooked, particularly in public health. Part of the reluctance toward lower-level metrics is understandable: For policy-makers, disaggregation increases detail, but reduces comparability. Although we refrain from prescribing a uniform solution, we urge leaders and researchers to carefully determine the scale on a case-by-case basis. As the COVID-19 pandemic demonstrated, global metrics are inadequate because they rely on highly aggregated data. Disaggregation to at least the PHC level can strengthen their precision and relevance. However, this improvement comes with a limitation: greater granularity often entails an inherent trade-off with policy scalability. As Fraser et al note, indicators that are “too local” are difficult to compare, whereas “over-aggregated” metrics risk being “detached from reality.”^46^ While traditional top-down approaches allow for statistical rigor and expert analysis,^75^ they often “exclude and marginalize the reality of the grassroots population,”^45^ rendering them ineffective for mobilizing community compliance during crises. Empirical findings suggest that populations only engage with indicators “if they relate to what they value.”^36^ Therefore, advancing pandemic readiness requires a hybrid approach: using co-production to ensure indicators capture local reality—avoiding the “social crises” that stem from ignored local sentiments^75^— while mapping these insights to broader policy domains to facilitate high-level decision-making.

 For example, the World Health Organization (WHO) has developed the first PHC measurement framework that guides a step-wise approach to the development and optimisation of indicators relevant to local needs and communities, including subnational priorities. Indeed, the report lists 48 indicators, referred to as tier 2 indicators, for PHC monitoring that it describes as indicators that are either infeasible to collect in most settings or require further methodological development.^76^ This nevertheless requires further methodological development and innovation, otherwise there is a risk of measuring the experts’ reality and values, not those of the local population, as facts and values are highly correlated in social indicator development.

 Secondly, a conflict exists between the perspectives of experts and the lived experiences of communities. This conflict, which is also evident between academics’ and practitioners’ perspectives, reflects a deeper ontological divide. However, scale adjustments alone cannot resolve this issue. Three solutions were identified in our review. Firstly, communities can actively participate in selecting the final indicators even if a top-down approach initiates the indicator development process, which allows for negotiation and/or approval by the lay public. Secondly, a more comprehensive approach involves co-constructing indicators with communities from beginning to end, by identifying, prioritising, designing, implementing, and evaluating them. This process is laborious and requires repeated adjustment. Thirdly, communities may independently generate their own quantification to which conventional statistical methods can be applied. Beyond one conference abstract,^65^ no further texts were available, and this idea merits further investigation. Overall, the evidence suggests that integrating experts’ “scientific facts” with communities’ “extended facts” offers a balanced solution, as has been repeatedly advocated for some time.^38,73,74,77^

 Thirdly, indicators are powerful political instruments. Conventional indicators are often expected to remain loyal to those in power. In contrast, developing indicators with substantial input from community members ensures that they reflect the lived experiences and needs of those communities. This bottom-up approach empowers individuals by providing a platform for their voices and concerns, ultimately leading to more relevant and effective indicators and policies. As some have argued, those who control the indicators effectively shape the narrative and, by extension, the outcomes.^70^

 However, GRIs can also become instruments of power. In her seminal essay, “Can the Subaltern Speak?” Gayatri Chakravorty Spivak examined the barriers that “subaltern” populations—especially women in the Global South—encounter when attempting to have their perspectives recognised and adequately interpreted within prevailing Western discourse frameworks.^15^ She critiqued both colonial and post-colonial intellectuals for failing to adequately represent the subaltern, suggesting that their attempts to give voice to the marginalised often result in further silencing them. Spivak’s main argument revolves around the idea that the true representation of subalterns requires more than just allowing them to speak; it necessitates an acknowledgement of the inherent biases and structures that shape and often distort their voices. Designed to reflect the specific needs and contexts of local populations, GRIs can either empower these communities or risk creating “docile voices” that conform to predetermined narratives. As Spivak critiques, while these indicators have the potential to advocate for the freedom and agency of marginalised groups by incorporating their perspectives and knowledge, they must be carefully managed to avoid replicating the same power dynamics. Ensuring that GRIs genuinely reflect the voices and priorities of subalterns requires ongoing vigilance against the biases and assumptions of those implementing these measures. When designed and utilised thoughtfully, GRIs can be a powerful tool for genuine representation and advocacy, but they must be critically examined to ensure they do not inadvertently perpetuate the silencing of marginalised voices.

 Lastly, local communities often possess profound and intimate knowledge of local contexts, circumstances, and issues that exceeds those of external researchers. Fundamentally, incorporating grassroots voices into the indicators addresses concerns of transparency while ensuring accountability and legitimacy.^40^ Furthermore, local communities are the ones that advocate for the indicators they trust. Thus, indicators that exclude grassroots voices are likely to face opposition from such communities. Empirical evidence suggests that individuals engage with indicators only when they resonate with their values, a phenomenon that can be substantiated through personal experience.^36^ One possible limitation of this approach to indicator development is that the mere origin of voices from the populace does not inherently endow them with unimpeachable authority. Indeed, a bottom-up approach reveals its limitations in contexts where local sentiments, beliefs, and values have contributed to social crises that necessitated the development of indicators in the first instance.^75^

 We believe that the strength of this review lies in its methodological rigour and cross-disciplinary approach, which reduces the risk of overlooking critical issues in indicator development, which can arise when a review is limited in depth and breadth to a single disciplinary tradition. By building on existing gaps rather than starting from scratch, our results are likely to be generalisable. We did not restrict our review to texts from isolated fields, which enabled us to identify the overarching patterns. The diversity of the authors of this review and their perspectives further reinforces this observation, at least from our perspective. However, a key limitation of our review is the variation in terminology across studies and the heterogeneity of populations and samples, which complicates comparisons and synthesis. This heterogeneity may have influenced our inclusion criteria, and subjective judgments are an inherent risk in such reviews. The four grey literature sources included in this review may also have biased our findings.

## Conclusions

 Disaggregating indicators at the grassroots level enhances their accuracy and relevance, thereby mitigating the risk of over-aggregation. However, this approach risks conflating the physical characteristics of grassroots environments with the experiences and perceptions of populations living within them. Relying solely on the grassroots level makes the interpretation increasingly complex. It is important to acknowledge the potential for conflicting realities; GRIs can counteract the bias inherent in developing metrics based solely on scientific assumptions or beliefs about what should be measured. Indicators developed through this approach gain legitimacy from the public’s perspective, empower local populations, and minimise the risk of unintended consequences. As highlighted throughout this review, top-down indicators provide comparability and oversight but often overlook local realities, while bottom-up indicators recover those realities but can sacrifice comparability. Recognising the complementary strengths and limitations of each approach is essential, and the most effective systems align indicators with purpose, context, and voice.

 Building on the gaps identified in this review, we suggest: (1) developing and trialling co-production protocols that give communities decision rights over indicator definitions, weights, and validation criteria; and (2) conducting “power audits” of GRIs (representation, exclusion risks, funder dependencies, and positionality disclosures) to prevent the production of docile voices.

## Disclosure of artificial intelligence (AI) use

 Not applicable.

## Ethical issues

 Not applicable.

## Conflicts of interest

 Authors declare that they have no conflicts of interest.

## Supplementary file


Supplementary file 1. Search Strategy.
