# Supplementary material for: Exploring Grassroots Indicators for Pandemic Prevention, Preparedness, and Response: A Systematic Narrative Review
Source: Int J Health Policy Manag. 2025 Dec 27;14:8886. doi: 10.34172/ijhpm.8886 (PMC12958240; doi:10.34172/ijhpm.8886)
Supplement: Supplementary file 1 — Search Strategy. [file ijhpm-14-8886-s001.pdf]

Article title: Exploring Grassroots Indicators for Pandemic Prevention, Preparedness, and Response: A Systematic Narrative Review

Journal name: International Journal of Health Policy and Management (IJHPM)

Authors' information: Million Tesfaye Eshete[1], Pami Shrestha[2], Charmaine Ang[3], José María Valderas[4], David L. Heymann[5], Anders Nordström[6], Kelley Lee[7], Alex Cook[4], Clare Wenham[8], Pablo Perel[5], J. Jaime Miranda[9,10], Alberto L. Garcia-Basteiro[11,12], Helen Clark[13], Helena Legido-Quigley[3,14], Eivind Engebretsen[1]*

[1]Centre for Sustainable Healthcare Education, Faculty of Medicine, University of Oslo, Oslo, Norway.

[2]National University Health System, Singapore, Singapore.

[3]Imperial College London, London, UK.

[4]Saw Swee Hock School of Public Health, National University of Singapore, Singapore, Singapore.

[5]London School of Hygiene and Tropical Medicine, London, UK.

[6]Department for Global Public Health, Karolinska Institutet and Centre for Resilent Health, Stockholm School of Economics, Stockholm, Sweden.

[7]Faculty of Health Sciences, Simon Fraser University, Vancouver, BC, Canada.

[8]London School of Economics and Political Science, London, UK.

[9]CRONICAS Center of Excellence in Chronic Diseases, Universidad Peruana Cayetano Heredia, Lima, Peru.

[10]Sydney School of Public Health, Faculty of Medicine and Health, The University of Sydney, Sydney, NSW, Australia.

[11]Centro de Investigação Em Saúde de Manhiça (CISM), Manhica, Mozambique.

[12]Barcelona Institute for Global Health (ISGlobal), Barcelona, Spain.

[13]The Helen Clark Foundation, Auckland, New Zealand.

[14]George Institute for Global Health UK, London, UK.

***Correspondence to:** Eivind Engebretsen; Email: eivind.engebretsen@medisin.uio.no

Citation: Eshete MT, Shrestha P, Ang C, et al. Exploring grassroots indicators for pandemic prevention, preparedness, and response: a systematic narrative review. Int J Health Policy Manag. 2025;14:8886. doi:10.34172/ijhpm.8886

Supplementary file 1. Search Strategy

Methods and analysis

Search strategy.

The following section details the search strategy we followed during the review process. We have followed the good practices for building search strategies for systematic reviews as recommended elsewhere[1,2]. Briefly we have: Identified the relevant keywords, phrases, and synonyms related to grassroots indicators that are appropriate to answer our research question.

1. We used controlled vocabulary customized to each database (MeSH, Headings, Subject areas, Categories) and free-text terms.
2. We have also considered variations in spelling, abbreviations, and different word forms
3. We have finally combined search terms with Boolean operators. As appropriate truncation, wildcard and adjacency operators were used.

Searching terms

1. (grassroot* adj4 indicator*)
2. (community-level adj4 indicator*)
3. (participatory adj4 indicator*)
4. (local-level adj4 indicator*)
5. (citizen-generated adj4 indicator*)
6. (bottom-up adj4 indicator*)
7. exp "Community-Based Participatory Research"
8. exp "Citizen Science"
9. or/1-4
10. or/1-8

Databases and pilot search results

Medline (Ovid)

#1

(grassroot* adj4 indicator*) OR (community-level adj4 indicator*) OR (participatory adj4 indicator*) OR (local-level adj4 indicator*) OR (citizen-generated adj4 indicator*) OR (bottom-up adj4 indicator*) OR (exp "Community-Based Participatory Research") OR (exp "Citizen Science")

# of articles search returned: 6,134

#2

indicator.mp. or "Indicators and Reagents"/

# of articles search returned: 238736

#3

#1 and # 2:

# of articles search returned: 67

#4

(grassroot* adj4 indicator*) OR (community-level adj4 indicator*) OR (participatory adj4 indicator*) OR (local-level adj4 indicator*)

# of articles search returned: 130

#5

2 AND 4

# of articles search returned:38

<u>Embase (Ovid):</u>

#1

(grassroot* adj4 indicator*) OR (community-level adj4 indicator*) OR (participatory adj4 indicator*) OR (local-level adj4 indicator*) OR (citizen-generated adj4 indicator*) OR (bottom-up adj4 indicator*) OR (exp "Community-Based Participatory Research") OR (exp "Citizen Science")

# of articles search returned:7,999

#2

indicator/

# of articles search returned: 1711

#1 and # 2:

# of articles search returned: 92

#3
(grassroot* adj4 indicator*) OR (community-level adj4 indicator*) OR (participatory adj4 indicator*) OR (local-level adj4 indicator*)

# of articles search returned:123

#4

#2 AND #3

# of articles search returned: 32

CINAHL:

#1

(grassroot* N4 indicator*) OR (community-level N4 indicator*) OR (participatory N4 indicator*) OR (local-level N4 indicator*) OR (citizen-generated N4 indicator*) OR (bottom-up N4 indicator*) OR ("Community-Based Participatory Research") OR ("Citizen Science")

# of articles search returned: TX =2, 398; Title =714; Abstract=2003

#2

AB ((grassroot* N4 indicator*) OR (community-level N4 indicator*) OR (participatory N4 indicator*) OR (local-level N4 indicator*) OR (citizen-generated N4 indicator*) OR (bottom-up N4 indicator*) OR ("Community-Based Participatory Research") OR ("Citizen Science")) AND AB Indicator

# of articles search returned: 84

#3

AB (grassroot* N4 indicator*) OR (community-level N4 indicator*) OR (participatory N4 indicator*) OR (local-level N4 indicator*)

# of articles search returned: 55

#4

AB Indicator

# of articles search returned:74457

#5

#4 AND #3

# of articles search returned: 55

Scopus:

TITLE-ABS-KEY (("grassroot*" OR "community-level" OR "participatory" OR "local-level" OR "citizen-generated" OR "bottom-up" OR "community-based participatory research" OR "citizen science"))

Number of articles search returned: 247,193

TITLE-ABS-KEY (Indicator)

# of articles search returned: 1,035,514

(TITLE-ABS-KEY (("grassroot*" OR "community-level" OR "participatory" OR "local-level" OR "citizen-generated" OR "bottom-up" OR "community-based participatory research" OR "citizen science"))) AND (TITLE-ABS-KEY (indicator))

# of articles search returned: 8,272

(TITLE-ABS-KEY ("grassroot*" OR "community-level" OR "participatory" OR "local-level") AND TITLE-ABS-KEY (indicator))

# of articles search returned: 6,716

TITLE-ABS-KEY (grassroot* AND indicator* OR community-level AND indicator* OR participatory AND indicator* OR local-level AND indicator*)

# of articles search returned: 241

<u>Web of Science (WOS):</u>

AB=(("grassroot*" OR "community-level" OR "participatory" OR "local-level" OR "citizen-generated" OR "bottom-up" OR "Community-Based Participatory Research" OR "Citizen Science"))

# of articles search returned:: 141, 753

(AB=("grassroot*" OR "community-level" OR "participatory" OR "local-level" OR "citizen-generated" OR "bottom-up" OR "Community-Based Participatory Research" OR "Citizen Science")) AND AB=(indicator)

# of articles search returned: 4,917

Searching in Abstracts- AB

#1

AB=(("grassroot*")) AND AB=(indicator)

# of articles search returned: 110

#2

AB=(("grassroot*" OR "community-level")) AND AB=(indicator)

# of articles search returned: 1,354

#3

AB=(("grassroot*" OR "community-level" OR "participatory")) AND AB=(indicator)

# of articles search returned: 2,986

#4

AB=(("grassroot*" OR "community-level" OR "participatory" OR "bottom-up")) AND AB=(indicator)

# of articles search returned: 3,800

#5
AB=(("grassroot*" OR "community-level" OR "participatory" OR "local-level" OR "bottom-up")) AND AB=(indicator)

# of articles search returned: 4743

#6

AB=(("grassroot*" OR "community-level" OR "participatory" OR "local-level" OR "citizen-generated" OR "bottom-up")) AND AB=(indicator)

# of articles search returned: 4, 746

#7

AB=(("grassroot*" OR "community-level" OR "participatory" OR "local-level" OR "citizen-generated" OR "bottom-up" OR "Community-Based Participatory Research")) AND AB=(indicator)

# of articles search returned: 4, 917

#8

Searching in Ttitles- TI

(TI=("grassroot*" OR "community-level" OR "participatory" OR "local-level" OR "citizen-generated" OR "bottom-up" OR "Community-Based Participatory Research" OR "Citizen Science")) AND TI=(indicator)

# of articles search returned: 142

#9

(TI=("grassroot*" OR "community-level" OR "participatory" OR "local-level" OR "citizen-generated" OR "bottom-up" OR "Community-Based Participatory Research")) AND TI=(indicator)

# of articles search returned: 136

#10

(TI=("grassroot*" OR "community-level" OR "participatory")) AND TI=(indicator)

# of articles search returned: 94

#11

#2 AND #10

# of articles search returned: 64

#12

(grassroot* indicator*) OR (community-level indicator*) OR (participatory indicator*) OR (local-level indicator*) (Abstract) and (grassroot* indicator*) OR (community-level indicator*) OR (participatory indicator*) OR (local-level indicator*) (Title)

# of articles search returned: 81

<u>PsychINFO:</u>

#1

("grassroot*" or "community-level" or "participatory" or "local-level" or "citizen-generated" or "bottom-up" or "Community-Based Participatory Research" or "Citizen Science")

Number of articles search returned: 32, 526

#2

#indicator

# of articles search returned: 29, 770

#3

("grassroot*" or "community-level" or "participatory" or "local-level" or "citizen-generated" or "bottom-up" or "Community-Based Participatory Research" or "Citizen Science")

# of articles search returned: 32526

#4

("grassroot*" or "community-level" or "participatory" or "local-level" or "citizen-generated" or "bottom-up")

# of articles search returned: 32273

#5
("grassroot*" or "community-level" or "participatory")

# of articles search returned: 32273

#6

#2 and # 5:

# of articles search returned: 131

#7

("grassroot*" or "community-level")

6757

#8

#2 AND 7

# of articles search returned: 58

#9

("grassroot*" or "community-level" or "participatory" or "local-level")

# of articles search returned: 25084

#10

#2 AND 9

# of articles search returned: 158

References

1. McGowan J, Sampson M, Salzwedel DM, Cogo E, Foerster V, Lefebvre C. PRESS Peer Review of Electronic Search Strategies: 2015 Guideline Statement. *Journal of Clinical Epidemiology.* 2016;75:40-46. doi:https://doi.org/10.1016/j.jclinepi.2016.01.021

2. Aromataris E, Riitano D. Constructing a search strategy and searching for evidence. *Am J Nurs.* 2014;114(5):49-56.
